# Tobacco use induces anti-apoptotic, proliferative patterns of gene expression in circulating leukocytes of Caucasian males

**DOI:** 10.1186/1755-8794-1-38

**Published:** 2008-08-18

**Authors:** Peter C Charles, Brian D Alder, Eleanor G Hilliard, Jonathan C Schisler, Robert E Lineberger, Joel S Parker, Sabeen Mapara, Samuel S Wu, Andrea Portbury, Cam Patterson, George A Stouffer

**Affiliations:** 1 Carolina Cardiovascular Biology Center, University of North Carolina at Chapel Hill, Chapel Hill, USA; 2 Division of Cardiology, School of Medicine, University of North Carolina at Chapel Hill, Chapel Hill, USA; 3 School of Medicine, Duke University, Durham, USA; 4 Expression Analysis Inc., Durham, USA

## Abstract

**Background:**

Strong epidemiologic evidence correlates tobacco use with a variety of serious adverse health effects, but the biological mechanisms that produce these effects remain elusive.

**Results:**

We analyzed gene transcription data to identify expression spectra related to tobacco use in circulating leukocytes of 67 Caucasian male subjects. Levels of cotinine, a nicotine metabolite, were used as a surrogate marker for tobacco exposure. Significance Analysis of Microarray and Gene Set Analysis identified 109 genes in 16 gene sets whose transcription levels were differentially regulated by nicotine exposure. We subsequently analyzed this gene set by hyperclustering, a technique that allows the data to be clustered by both expression ratio and gene annotation (*e.g*. Gene Ontologies).

**Conclusion:**

Our results demonstrate that tobacco use affects transcription of groups of genes that are involved in proliferation and apoptosis in circulating leukocytes. These transcriptional effects include a *repertoire *of transcriptional changes likely to increase the incidence of neoplasia through an altered expression of genes associated with transcription and signaling, interferon responses and repression of apoptotic pathways.

## Background

Gene expression profiling has become a powerful approach to the study of molecular pathophysiology and is a potentially useful diagnostic tool in multiple fields [1]. Oncologists have applied gene expression profiling to predict breast cancer aggressiveness [2], and microarray-driven approaches have been used to analyze cardiovascular diseases such as hypertension, heart failure, cardiac rejection, and atherosclerosis [3-5]. Ideally, gene expression profiling is performed on the specific cell type and tissue of interest, *i.e*. the tumor, myocardium, or atheroma. However, sampling target tissues from humans is often problematic, and data derived from tissues not routinely available to clinicians limits the diagnostic utility of this approach.

For the study of biological processes that involve an inflammatory response, gene expression profiles can be obtained from circulating leukocytes [6]. Due to the ease of sampling, gene expression profiling of circulating leukocytes has been applied to the study of cancer [7], atherosclerosis [8,9], and systemic lupus erythematosus [10]. These studies demonstrate the utility of transcriptional analysis of peripheral blood in the study of disease states having a systemic, inflammatory component.

Tobacco use, whether by smoking or chewing, is associated with the development of many diseases. People who smoke more than 20 cigarettes per day have a 3–6 fold increased incidence of myocardial infarction [11] and increased overall rates of cardiovascular mortality compared to those who have never smoked [12]. The risk of developing lung cancer is 20-fold increased in cigarette smokers [8], and smokers are at increased risk of developing chronic obstructive pulmonary disease, multiple cancers (*e.g*. esophageal, bladder, and leukemia), pneumonia, osteoporosis, and periodontal disease [13]. Despite these major adverse health effects, more than 20% of American adults identify themselves as active smokers [14].

The mechanistic link between tobacco smoking and related diseases remain incompletely understood. To date, there have been numerous reports analyzing the effect that exposure to cigarette smoke has on the gene expression profiles of various cell types [15-22]. However, despite this detailed analysis, very little consensus amongst findings has been reported, even when the same cell type has been studied [16]. This lack of significant overlap in conclusions may be the result of the considerable heterogeneity in methodology as well as the relatively small (on average 5–10 test subjects) sample populations in each study. Furthermore, many of these reports rely on the *in vitro *exposure of cells to cigarette smoke condensate, raising the obvious issue of physiological relevance amongst these various studies.

Here we report a novel method for analyzing the *in vivo *effects of tobacco use on gene expression in circulating leukocytes. The purpose of this study is not to identify biomarkers associated with tobacco use; rather, our approach is aimed at identifying changes in genes and gene sets that result from tobacco use and applying this information to identify potential cellular pathways associated with tobacco-dependent pathology. Our results indicate that tobacco use affects pathways that control cell death, response to stress, macromolecular metabolism and the inflammatory cascade, providing new insights into the systemic effects of smoking that may underlie tobacco-related diseases.

## Methods

### Subject Population

Subjects between the ages of 18 and 50 years (inclusive) referred to UNC Hospitals were considered for enrollment in this University of North Carolina Institutional Review Board-approved study (IRB 04-MED-471). Exclusion criteria included current cancer treatment, pregnancy, lymphoma, leukemia, chronic immunosuppressive therapy, infection with HIV or HCV, history of solid organ transplant, and anemia (*i.e*. conditions which might alter peripheral blood counts or patterns of gene expression). After obtaining informed consent for a one-time blood donation, subjects were interviewed for pertinent medical information, including a detailed history of tobacco use, family history of heart disease and diabetes. Blood cell counts including a white blood cell differential analysis was performed to ensure consistency in cell subtype number between study populations.

### Blood Withdrawal and Processing

Blood (30 ml) was drawn early in the day from subjects fasted for at least 8 hours to minimize the signals associated with nutritional and diurnal cycles from the microarray data. Processing was begun within 15 minutes of the time of blood draw. Eight ml were collected into a tube containing EDTA and proteinase inhibitors (Becton, Dickinson and Co., Cockeysville, MD) to provide a sample of plasma for cotinine assays. The balance of blood was collected into Na-EDTA Vacutainer tubes (Becton, Dickinson and Co., Cockeysville, MD). Whole blood was treated with 10 volumes of carbonate-buffered 150 mM NH_4_Cl to lyse red blood cells. The remaining leukocytes were washed and concentrated by centrifugation [23,24]. RNA and DNA were recovered from leukocytes using a modified one-step acid guanidinium isothiocyanate-phenol-chloroform extraction (RNA-STAT60, Tel-Test, TX). RNA was subsequently post-purified using the RNeasy Mini-kit (Qiagen, Valencia, CA). RNA quantity, purity, and integrity were assessed by spectrophotometry and microcapillary electrophoresis on an Agilent BioAnalyzer 2100. Complete processing of samples occurred within 2 hours, exceeding the standards set by the Consortium for Expression Profiles in Sepsis [25]. Plasma cotinine levels were determined by competitive ELISA using the Serum Cotinine Assay Kit (BioQuant; San Diego, CA) essentially as described by the manufacturer.

### Gene Expression Profiling

We utilized a "sample × reference" experimental design strategy in which RNA from each subject was hybridized to the microarray slide in the presence of labeled human reference RNA (UHRR, Stratagene, La Jolla, CA) [26,27]. Briefly, total RNA (500 ng) was used for gene expression profiling following reverse transcription and T-7 polymerase-mediated amplification/labeling with Cyanine-5 CTP. Labeled subject cRNA was co-hybridized to Agilent G4112A Whole Human Genome 44 K oligonucleotide arrays with equimolar amounts of Cyanine-3 labeled UHRR. Slides were hybridized and washed, then scanned on an Axon 4000b microarray scanner. The data were processed using GenePix Pro 6 software and entered into the UNC Microarray Database [28].

### Quantitative Real Time Polymerase Chain Reaction (qRT-PCR) analysis

Three hundred nanograms of total RNA were reverse transcribed using the iScript Synthesis cDNA Kit (Biorad, Hercules, CA). Real-time PCR reactions were performed using either the Roche Universal Probe Library (Roche Diagnostics, Mannheim, Germany) or pre-validated Taqman^® ^assays (Applied Biosystems, Framingham, MA). Primers and probes for the indicated human transcripts were designed using Probe Finder (version 2.41, Roche Diagnostics, Mannheim, Germany): *CDKN1C *(left primer GAGCGAGCTAGCCAGCAG, right primer GCGACAAGACGCTCCATC, probe #77); *CX3CR1 *(left primer CTCTGGCTTCTGGGTGGAG, right primer AGACCACGATGTCCCCAATA, probe #30); *SASH1 *(left primer CAGATCCGGGTGAAGCAG, right primer GAGTCCACCACTTGGAATCG, probe #38); *RPS29 *(left primer CCAAGAACTGCAAAGCCATC, right primer GGCATTGGTGACTCTGATGA, probe #26); and *18S *(left primer GGAGAGGGAGCCTGAGAAAC, right primer TCGGGAGTGGGTAATTTGC, probe #40). *PTGDR *and *HRASLS3 *were measured using Taqman^® ^assays Hs00235003_m1 and Hs00272992_m1, respectively. Real-time PCR reactions were performed using the ABI PRISM^® ^7900 sequence detection system, software, and reagents. Relative changes in gene expression were calculated using the delta Ct method using ribosomal *18S *to normalize RNA input. Statistical significance was determined using the Student's *t *test. *P *values less than 0.05 were considered significant.

### Statistical Methods

Microarray data were normalized *via *the loess local intensity normalization [7,29], and probes were filtered for features having a normalized intensity of < 30 aFU in either channel. Probes were removed if < 70% of the data were present across all samples. Missing data points were imputed using the k nearest-neighbors algorithm (k = 10). 18,375 probes passed these filters, and were subsequently used for analysis. Scripts written in the R Statistical Language and Environment ("R"; Version 2.2.1, build r36812, release date 2005-12-20.) and Perl (ActiveState Perl 5.8.1, build 807, release date 2003-11-6) were used to standardize (μ = 0, σ = 1) each sample in the data set.

### Statistical Analysis of Microarrays (SAM)

Lists of differentially expressed genes were identified using the statistical analysis of microarray algorithm [30-32] (SAM, Version 2.21, release date 2005-8-24; typical false discovery rate of approximately 10%). Unsupervised, semi-supervised, and supervised clustering analysis was performed on gene lists essentially as described [33] using Cluster, version 2.11[34]. Heat maps of cluster analyses were visualized with JavaTreeView, version 1.0.12 [35,36].

### Gene Set Analysis (GSA)

GSA [37,38] was performed using the Molecular Signatures Database (MSigDB) [39] to identify gene set activity associated with cotinine levels. Mapping to gene ontology categories (GO) [40] and identification of putative transcription factor binding sites was performed on gene lists using the GATHER web-based analysis environment [41-43] using the TRANSFAC V7.0 (public) database [44-47].

### Hyperclustering

A median-centered gene list was used for cluster analysis to identify relationships between subject samples (arrays). The clustering file was then used as the basis for a new pre-clustering file to incorporate gene annotation data. Genes were assigned to GO and TRANSFAC categories using the GATHER web interface [42]. Categories showing statistical enrichment (p value < 0.01) were identified, and each gene in the pre-clustering file was annotated as to its membership in the appropriate category. The TRANSFAC predictions of transcription factor binding sites were designated in the pre-clustering file by the value representing the median-centered mean fold change expressed as the Log_2 _of the ratio of each sample to the reference for each gene. This method of indicating membership was chosen to reflect a relationship between expression level (as measured by microarray) and presence or absence of transcription factor binding sites. Gene membership in GO categories was indicated by a binary value of either 1.00 or 0.00 as a placeholder for the expression ratio. Blue color was added after the fact to heat maps indicating Gene Ontology membership to avoid confusion with expression values. The annotated pre-clustering file was then clustered on only the Y axis (genes) to preserve relationships among arrays. This technique, which we have designated "Hyperclustering," allows both the gene expression data and various other forms of annotation to be represented as a single heat map, effectively illustrating functional relationships among genes.

## Results and discussion

### Subject Selection for Gene Expression Analysis

Initial analysis of the gene transcription data from a cohort of 171 individuals revealed strong signals related to the race and gender of the subject (unpublished observations). Similar signals have been described in other microarray experiments. These signals can hinder attempts to identify signals related to the biological effect being studied [48]. For this reason, we selected the largest cohort in our dataset (Caucasian males) to maximize the statistical power of our analysis. We adopted a case-control approach to our study design and data analysis. Selected subject demographics are presented in Table 1.

**Table 1:** Selected demographics of study subjects.

		**Low Cotinine**	**High Cotinine**
Number of subjects		38	24
Mean Age ± SD		47 ± 9	46 ± 5
COPD		2 (5.3%)	4 (16.7%)
Diagnosis of Diabetes (Number (% of total))	*Any	13 (34%)	2 (8.3%)
	Type 1	2 (5%)	1 (4%)
	Type 2	11 (29%)	1 (4%)
CAD Family History		20 (53%)	15 (63%)
Hyperlipidemia		24 (63%)	16 (67%)
Automated Differential Blood Count	White Blood Cells (× 10^9 ^/L ± SD)	8.42 ± 2.67	9.00 ± 2.41
	Neutrophils (× 10^9 ^/L ± SD)	5.67 ± 2.18	5.76 ± 1.94
	Lymphocytes (× 10^9 ^/L ± SD)	1.90 ± 0.68	2.31 ± 0.74
	Monocytes (× 10^9 ^/L ± SD)	0.42 ± 0.18	0.46 ± 0.21
	Basophils (× 10^9 ^/L ± SD)	0.06 ± 0.04	0.06 ± 0.04
	Eosinophils (× 10^9 ^/L ± SD)	0.22 ± 0.18	0.26 ± 0.14
	Platelets (× 10^9^/L ± SD)	252.42 ± 73.97	250.67 ± 56.06

CAD = Coronary Artery Disease, SD = Standard Deviation, L = liter, fL = femtoliter, dL = deciliter, G = gram, pG = picogram, COPD = Chronic Obstructive Pulmonary Disease. For Student's T-test, automated cell counting values were recalculated as values per gram or liter, and log2 normalized prior to determination of p-Value.

* Fisher's Exact Test shows significant differences between low and high cotinine at p = 0.0315 (2-tail)

### Tobacco Use Determination

**Figure 1:**
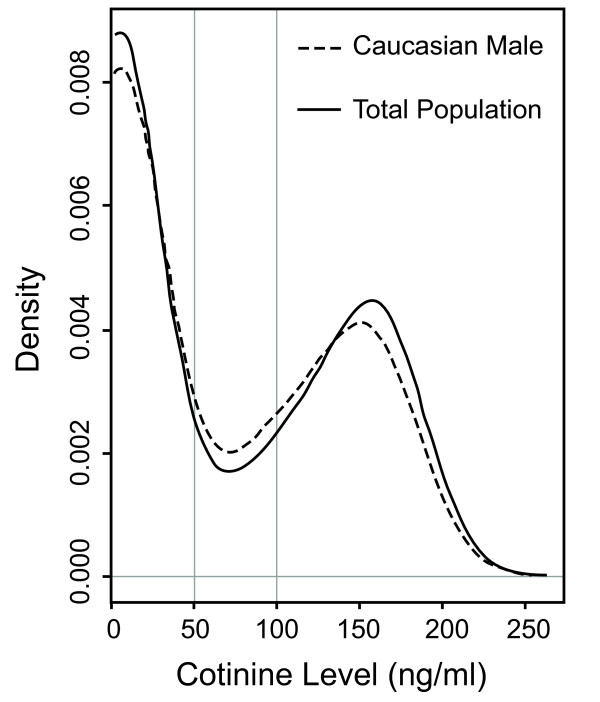
**Histogram of plasma cotinine concentration**. Distribution of plasma cotinine levels in the total population as well as in the Caucasian male sub-population are demonstrated. Vertical lines represent selected cut-offs for definitions of tobacco users and non-users.

Self-reported tobacco use history is notoriously inaccurate [49-51]. For purposes of this study, we defined tobacco use status by the subject's plasma cotinine concentration. Cotinine, the principle metabolite of nicotine, is a reliable surrogate marker of tobacco use [52,53]. It has a plasma half-life of approximately 24 hours (as opposed to nicotine's *in vivo *half-life of 30 minutes) and tends to reach steady state levels that vary by only 15%–20% in people with regular smoking habits [52]. As seen in Figure 1, the distribution of plasma cotinine is similar in both the Caucasian male subpopulation under study and a larger cohort of 171 subjects, with strong bimodal peaks near 0 ng/mL and 150 ng/mL. Cutoffs of plasma cotinine for the definition of active tobacco users and non-users were set at > 100 ng/mL and < 50 ng/mL, respectively, based on previously reported values [52,53].

Using these criteria, 24 subjects were classified as tobacco users and 38 as non-tobacco users, with 5 subjects having cotinine levels that fell between 50 and 100 ng/mL. These 5 intermediate subjects were removed from further consideration. Comparing each subject's plasma cotinine values with their self-reported tobacco use status revealed overall consistent results (*i.e*. a high cotinine value for subjects who self-reported that they were active tobacco users). Nevertheless, there were notable exceptions. Seven subjects reported that they were non-tobacco users, yet had plasma cotinine levels > 100 ng/mL. Errors in this dimension could be explained by subject misrepresentation or failure of the subjects to disclose nicotine replacement therapy as part of a smoking cessation plan (use of nicotine patches or gum). Interestingly, 3 subjects identified themselves as active smokers, yet had very low plasma cotinine levels. Rapid metabolism of nicotine, smoking of a small number of cigarettes daily, or the use of extremely low-nicotine smoking products could all account for this discrepancy. This discrepancy in self-reported tobacco use and plasma cotinine levels did not appreciably alter the results of our studies (data not shown). All subjects were categorized based only on plasma cotinine levels only. The 2 subject groups will henceforth be referred to as "high cotinine" (*i.e*. tobacco users) and "low cotinine" (*i.e*. non-tobacco users). Using this criterion, those subjects reporting to be "smokers" but who had low plasma cotinine levels were included in the low cotinine group while subjects with high cotinine levels who denied smoking were included in the high cotinine group. To ensure that patient co-morbidities did not influence the gene expression profile, we performed principal components analysis (PCA) on the expression values of genes identified in this paper using the combined significant gene list and visualized in the context of COPD, diabetes, CAD class, and smoking status (Additional File 1). As expected, the top component of variation appears to be associated only with smoking status.

### Transcriptional Signals of Tobacco Use

The subjects were stratified based upon the results of the cotinine assay, and differential gene expression was determined by SAM. We identified 38 genes as being differentially expressed (8 genes up-regulated, 30 genes down-regulated in the high-cotinine group) at an 11.7% FDR (Table 2). Notable among this list were genes involved in apoptosis, cell cycle regulation, and oncogenesis.

**Table 2:** Differentially expressed genes identified by SAM analysis.

**Down-regulated in High Cotinine Subjects**				
**Gene Symbol**	**Gene Name**	**Accession Number**	**Agilent Probe ID**	**Mean FC**
HRASLS3	HRAS-like suppressor 3	NM_007069	A_23_P116414	1.5
CX3CR1	Chemokine (C-X3-C motif) receptor 1	NM_001337	A_23_P407565	1.3
GPR56	G protein-coupled receptor 56	NM_005682	A_23_P206280	1.3
PTGDS	Prostaglandin D2 synthase 21kDa (brain)	NM_000954	A_23_P146554	1.3
FLJ23262		BC043173	A_24_P20996	1.2
BRD1	Bromodomain containing 1	NM_014577	A_23_P166536	1.2
BZRAP1	Benzodiazapine receptor (peripheral) associated protein 1	NM_004758	A_23_P152559	1.2
C1D	Nuclear DNA-binding protein	NM_173177	A_23_P67992	1.2
FLJ23262		BC043173	A_24_P20996	1.2
CTCF	CCCTC-binding factor (zinc finger protein)	NM_006565	A_24_P347704	1.2
DNAJB6	DnaJ (Hsp40) homolog, subfamily B, member 6	NM_058246	A_24_P63827	1.2
ENST00000320343		ENSG00000177197	A_24_P75688	1.2
FLJ35696		NM_207387	A_23_P368484	1.2
GNG2	Guanine nucleotide binding protein (G protein), gamma	NM_053064	A_32_P208403	1.2
HS6ST1	Heparan sulfate 6-O-sulfotransferase 1	AL831893	A_24_P8220	1.2
IKIP	IKK interacting protein	NM_201613	A_23_P53467	1.2
KLRK1	Killer cell lectin-like receptor subfamily K, member 1	NM_007360	A_23_P218058	1.2
MAF	V-maf musculoaponeurotic fibrosarcoma oncogene homolog (avian)	AF055376	A_24_P256219	1.2
MGC61571		NM_182523	A_24_P408740	1.2
MTCBP-1	Membrane-type 1 matrix metalloproteinase cytoplasmic tail binding protein-1	NM_018269	A_23_P148194	1.2
AL137798		NM_032723	A_23_P126486	1.2
OSBPL5	Oxysterol binding protein-like 5	NM_145638	A_23_P53081	1.2
PPP1CB	Protein phosphatase 1, catalytic subunit, beta isoform	NM_206877	A_23_P83414	1.2
PPP1R12B	Protein phosphatase 1, regulatory (inhibitor) subunit 12B	NM_002481	A_23_P201790	1.2
PPP2R2B	Protein phosphatase 2 (formerly 2A), regulatory subunit B (PR 52), beta isoform	NM_181678	A_23_P213620	1.2
SLC25A20	Solute carrier family 25 (carnitine/acylcarnitine translocase), member 20	NM_000387	A_23_P72025	1.2
SLC9A3R1	Solute carrier family 9 (sodium/hydrogen exchanger), isoform 3 regulator 1	NM_004252	A_23_P308519	1.2
SULF2	Sulfatase 2	NM_198596	A_23_P154605	1.2
YWHAQ	Tyrosine 3-monooxygenase/tryptophan 5-monooxygenase activation protein, theta polypeptide	NM_006826	A_24_P199905	1.2
PTGDR	Prostaglandin D2 receptor (DP)	NM_000953	A_23_P393777	1.1
				
**Up-regulated in High Cotinine Subjects**				
**Gene Symbol**	**Gene Name**	**Accesion Number**	**Agilent Probe ID**	**Mean FC**
SASH1	SAM and SH3 domain containing 1	NM_015278	A_23_P93442	1.4
BC107798		NM_003283	A_23_P56050	1.4
AL442066		AL442066	A_23_P123645	1.3
DNAPTP6	DNA polymerase-transactivated protein 6	NM_015535	A_23_P131255	1.3
C1GALT1	Core 1 UDP-galactose:N-acetylgalactosamine-alpha-R beta 1,3-galactosyltransferase	NM_020156	A_23_P252145	1.2
RGL1	Ral guanine nucleotide dissociation stimulator-like 1	NM_015149	A_23_P115417	1.2
CTMP	C-terminal modulator protein	NM_176853	A_23_P149375	1.2
LOC283174	Hypothetical protein LOC283174	NM_001001873	A_24_P904484	1.2

Visual inspection of the SAM-identified genes revealed that a number of differentially expressed genes are involved in the cell cycle control Gene Ontologies. *CTCF *was down regulated in the high cotinine group. Mutations in this gene have been associated with a variety of cancers [54]. Furthermore, *CTCF *plays an important role in the regulation and differentiation of human myeloid leukemia cells, adding another possible underlying mechanism of leukemiagenesis in tobacco users [55]. Conversely, we found that *SASH1 *(which is implicated in tumorogenesis of colorectal and breast cancer) was up regulated [56]. Interestingly, *CX3CR1 *was significantly down regulated in the high cotinine group. As *CX3CR1 *is up-regulated in atherosclerotic lesions [57], we expected it to be up-regulated in circulating leukocytes of tobacco users due to the increased incidence and severity of CAD in this population (reviewed by Njolstad [11]). However, Barlic, *et al*., showed that macrophage up-regulation of *CX3CR1 *leads to retention of those cells in vessel walls [57]. As the kinetics of the up-regulation of this gene are ill-defined, and it is not yet clear whether circulating monocytes differentially express *CX3CR1 *prior to tissue macrophage transformation, considerably more study will be necessary to elucidate what role it may play in the pathogenesis of smoking-related atherosclerotic disease.

Further analysis identified genes involved in apoptotic pathways. The pro-apoptotic genes *C1D*, *MTCBP-1*, *CTCF*, *IKIP*, *MAF*, and *YWHAQ *were all significantly down regulated in the high cotinine group. *C1D *(also known as *SUNCOR*) is representative of this group. *C1D *is a multi-functional nuclear protein with DNA-binding properties. When *C1D *is experimentally over-expressed it activates *DNA-PK*, inducing apoptosis [58]. On the other hand, the c-terminal modulator protein (*CTMP*, also known as *THEM4*) was significantly over-expressed in the high cotinine population. CTMP protein stimulates the phosphorylation of AKT/PKB, increasing glucose uptake and blocking apoptosis [22]. The relative mean fold change was modest for most of these genes (Table 2); nevertheless, in subjects with high plasma cotinine the overall expression pattern of these genes is anti-apoptotic compared to low cotinine subjects. The combination of increased cell cycle activity, resistance to apoptotic triggers, increased expression of oncogenes, and decreased expression of tumor suppressor genes in circulating leukocytes suggests a mechanism responsible for the low-level, systemic, increased risk of oncogenesis in patients who use tobacco products.

Testing for differential expression of individual genes does not take advantage of our knowledge of the underlying relationships. Therefore, additional power can be gained by testing for differential expression of gene sets that underlie a common biological process [37,38,59]. This idea motivated the development of techniques that pair local statistics of individual gene expression with global statistics based on membership in defined pathways and functional groups. One such algorithm, Gene Set Analysis (GSA), was implemented using the Molecular Signatures database (MSigDB). The GSA algorithm identified 16 gene sets at a p-value < 0.0001 and FDR of 0%. The top three MSigDB pathways were "Death Pathway," "Dac_IFN_Bladder_Up," and "Metastasis_Adenocarcinoma" (Table 3). Although many of the genes comprising these sets did not reach statistical significance individually, taken as a group they were highly significant. Genes related to apoptosis and type I interferon response were common elements in all of these pathways. Among genes involved in the MSigDB "Death Pathway," expression of *BIRC3 *and *TRAF2 *(anti-apoptotic genes) were up regulated while *CASP9*, *FADD*, and *STK17A *(pro-apoptotic genes) were down regulated in the high cotinine group. This overall expression pattern is indicative of an anti-apoptotic phenotype, which characterizes virtually all cancers. These observations suggest that transcriptional profiles associated with tobacco use may indicate pre-cancerous tendencies. The 71 genes present in the top 3 pathways (Table 3) were added to the list of 38 SAM-identified genes to enrich the gene list that was used for further analysis. This list of 109-pooled genes is available as Additional file 2.

**Table 3:** Summary of GSA.

**Gene Set Pathway**	**Description**	**P-value**	**FDR**
DEATHPATHWAY (c2:161)[71]	Genes involved in signaling via Fas and DR3, 4, and 5.	< 0.0001	0
METASTASIS_ADENOCARC_DN (c2:1553)[72]	Genes involved in metastasis of solid tumors.	< 0.0001	0
DAC_IFN_BLADDER_UP (c2:1304)[73]	Interferon responsive genes upregulated by DAC treatment.	< 0.0001	0

Top 3 gene sets (71 total genes) identified by GSA comparing gene expression profiles of subjects with high plasma cotinine versus low plasma cotinine, showing the names of differentially expressed gene sets as defined by Gene Set Enrichment Analysis [70] with accompanying p-value and FDR.

### Pattern Identification *via *the Hyperclustering Technique

**Figure 2:**
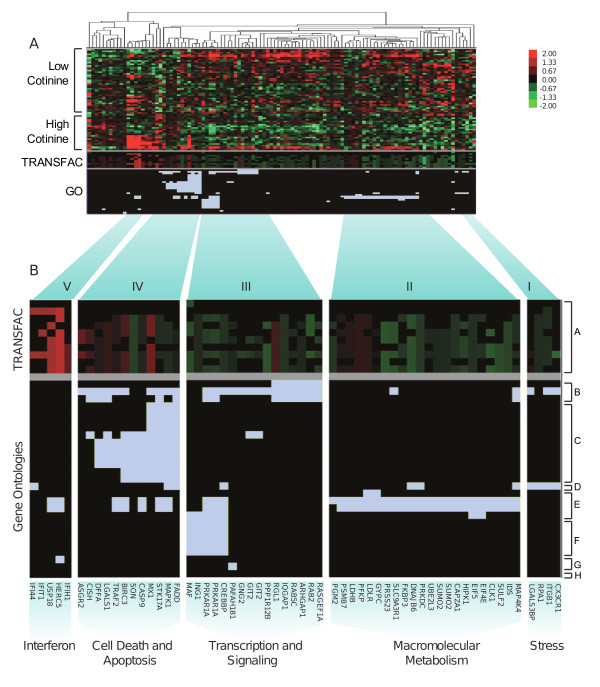
**Hyperclustering of cotinine responsive genes**. **A**. The 109 genes identified by SAM and GSA analysis in subjects with high versus low plasma cotinine levels were analyzed by hyperclustering. Clusters (top) were created by incorporating gene expression data with their corresponding TRANSFAC and Gene Ontology (GO) categories. Genes are represented in columns. Individual subject expression profiles (which clustered into 2 groups, high and low cotinine) and TRANSFAC categories are represented in rows and the relative expression of the genes is reflected as indicated in the color scale (upper right). Gene membership in GO categories (also represented in rows) is indicated by Carolina blue. **B**. Enlargement of the five functional groups identified by hyperclustering (bottom). The corresponding TRANSFAC and GO categories are indicated by groups A and B-H, respectively (see Table 4 for detailed category information).

Differentially expressed genes were hyperclustered (see Materials and Methods) and visualized (Figure 2) using the pooled gene list. The subjects with the highest mean levels of cotinine were clearly separated from the subjects with the lowest mean cotinine levels using this technique. Moreover, genes were clustered into functional groups based on their expression patterns, membership in Gene Ontologies (Table 4, labeled A-G), and presence of predicted transcription factor binding sites. This produced 5 physiologically relevant clusters. The '*Stress*' cluster is comprised of stress-responsive genes involved in signal transduction (*CX3CR1 *and *ITGB1*). The '*Macromolecular Metabolism*' cluster is made up of metabolic genes (*HIPK1*, *SUMO2*, *SULF2*, and *FKBP3*). The third cluster, '*Transcription and Signaling'*, contains genes associated primarily with G protein signaling and transcriptional regulation (*RASGEF1A*, *RAB2*, *ARHGAP1*, *PPP1R12B*, *CREBBP*, and *GNG2*). '*Cell Death and Apoptosis*' is a cluster of genes associated with apoptosis and its regulation. The fifth cluster, '*Interferon' *is defined by genes that potentially contain an interferon-stimulated response element-binding site or are responsive to type-1 interferons.

**Table 4:** Hyperclustered TRANSFAC and GO Category Annotations

Cluster	TRANSFAC Annotations			
A	V$POU3F2_02			
	V$ISRE_01: interferon-stimulated response element			
	V$DEAF1_01			
	V$E2F1_Q3_01			
	V$MAZR_01: MAZ related factor			
	V$KROX_Q6			
	V$E2F1DP1_01: E2F-1:DP-1 heterodimer			
	V$HNF1_Q6			
	V$E2F_Q3_01			
	V$E2F1_Q6: E2F-1			

Cluster	Common GO Parent Node	Gene Ontology	GO Level	GO Name
B	signal transduction [4] GO:0007165	GO:0007264	6	small GTPase mediated signal transduction
		GO:0007165	4	signal transduction
		GO:0007242	5	intracellular signaling cascade
C	programmed cell death [5] GO:0012501	GO:0006917	8	induction of apoptosis
		GO:0012502	7	induction of programmed cell death
		GO:0043068	6	positive regulation of programmed cell death
		GO:0043065	7	positive regulation of apoptosis
		GO:0050794	3*	regulation of cellular process
		GO:0016265	3*	death
		GO:0008219	4*	cell death
		GO:0012501	5	programmed cell death
		GO:0006915	6	apoptosis
		GO:0043067	5	regulation of programmed cell death
		GO:0042981	6	regulation of apoptosis
D	response to stress [4] GO:0006950	GO:0006950	4	response to stress
E	macromolecule metabolic process [4] GO:0043170	GO:0006493	9	O-linked glycosylation
		GO:0043170	4	macromolecule metabolism
		GO:0044260	5	cellular macromolecule metabolism
		GO:0019222	4*	regulation of metabolism
F	transcription [6] GO:0006350	GO:0006350	6	transcription
		GO:0045449	6	regulation of transcription
		GO:0019219	5*	regulation of nucleo-base, -side, -tide and nucleic acid metabolism
		GO:0006355	7	regulation of transcription, DNA-dependent
		GO:0006351	7	transcription, DNA-dependent
G	cell cycle process [6] GO:0022402	GO:0000082	7	G1/S transition of mitotic cell cycle
		GO:0000132	11	mitotic spindle orientation
H	mevalonate transport [8] GO:0015728	GO:0015728	8	mevalonate transport

* Node is not a child of the parent node for this group

The utility of the hyperclustering technique is readily apparent: a single image indicates the relationships among the genes, lending physiological relevance to a data set. A case in point is the '*Interferon' *cluster, comprised of genes that are strongly up regulated in approximately half of the subjects with the highest cotinine levels. The genes in this cluster (*IFI44*, *IFIT1*, *USP18*, and *HERC5*. Figure 2) are interferon responsive genes, and are members of the gene class forming the early response to type-I interferons, indicative of a cellular response to viral agents or very specific forms of genotoxicity. Our findings are consistent with those of Grumelli, *et al*. who demonstrated that lymphocytes isolated from lung samples of patients with smoking-related lung damage showed an increase in expression of multiple interferon-inducible proteins [60]. These results indicate that induction of interferon-dependent transcription pathways appear systemically in some tobacco users. Only half of the tobacco users have this expression pattern; the mechanisms of which are unknown, but worthy of future investigation. It is tempting to speculate that these patterns of systemic interferon-responsive induction identify a group of tobacco users who may develop early and severe disease. Longitudinal studies designed to track the patterns of gene expression over time in cohorts of tobacco users and non-users will be necessary to unambiguously determine the meaning of these observations.

### Real time PCR verification of differentially expressed genes

**Figure 3:**
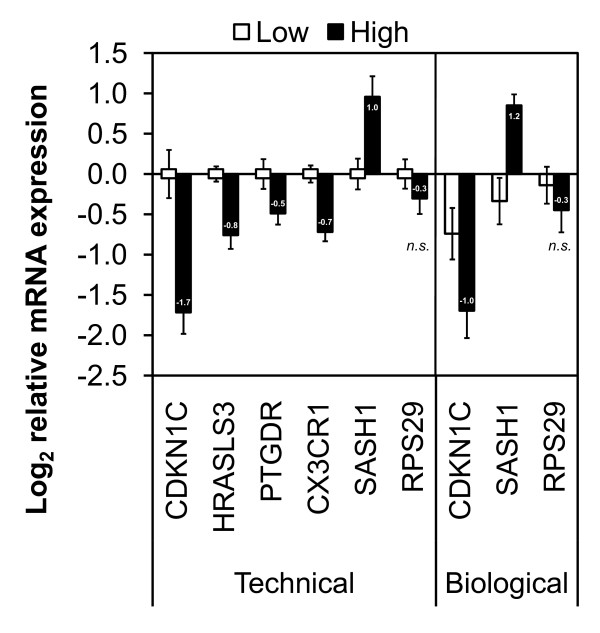
**Histogram of relative expression of selected genes using real time PCR**. *Technical *verification (left) of differentially expressed genes identified in the subject population (Caucasian males) by SAM/GSA (n = 20): *CDKN1C*, *HRASLS3*, *PTGDR*, *CX3CR1*, and *SASH1*. *Biological *verification (right) of two selected genes using independent samples not included in our subject population (Caucasian females, n = 10): *CDKN1C *and *SASH1*. Data is represented as the log base 2 relative change in gene expression (± standard error) and all expression normalized to low cotinine from the subject population samples (Caucasian males). The data labels represent the fold change in high versus low cotinine samples, all of which were statistically significant (*P *< 0.05). The fold change in the gene *RPS29 *was used as a negative control and was not significant (*n.s*.) between the high and low cotinine groups.

Quantitative real time PCR was used for both *technical *(microarray) and *biological *verification. Four genes selected from SAM and one gene from GSA: *CX3CR1*, *SASH1*, *HRASLS3*, *PTGDR*, and *CDKN1C*, respectively, were used for technical verification (Figure 3, left panel) on samples randomly selected from the low and high cotinine subject population (Caucasian males). The up or down regulation of these genes, irrespective of their method of identification (SAM or GSA) was consistent with the microarray analysis. Furthermore, the relative fold changes determined *via *quantitative real time PCR were either equal to or greater than the fold change measured by the microarray analysis, and significantly different between the low and high cotinine subjects (*P *< 0.05). Analysis using subjects excluded from the microarray analysis (Caucasian females) biologically validated the cotinine-dependent change in expression of two genes, *CDKN1C *and *SASH1 *(Figure 3, right panel). *RPS29 *was used as a negative control gene and was not found to be differentially expressed either by microarray or real time PCR analysis.

## Conclusion

In this study we demonstrated that groups of genes in circulating human leukocytes are affected by tobacco use *in vivo*. We identified genes and their relationships using a combination of testing individual genes (SAM), testing gene sets (GSA), and high throughput annotation (GATHER). Hyperclustering using Gene Ontologies and transcription factor binding sites associated with these genes illuminated the functional significance of the differentially regulated genes. The resulting gene expression spectra revealed novel and under-recognized molecular pathways in the pathophysiology of diseases commonly associated with tobacco use. Genomic signals in circulating leukocytes characteristic of cellular metabolism, transcription and signaling, apoptosis, response to stress, and the interferon response were all correlated with nicotine exposure. These results strongly suggest that tobacco use promotes a pro-carcinogenic environment, predisposing individuals to develop cancers in a variety of organ systems.

Interestingly, some genes that have previously been linked to smoking were not differentially expressed in our 2 subject groups [61-63]. For example, neither *CYP1B1 *(a cytochrome P450 enzyme playing an important role in chemical carcinogenesis) nor *SOD2 *(which destroys toxic radicals normally produced within cells) had an expression profile that differed significantly between high and low cotinine groups. Although several previous reports identified these genes as being affected by smoking, design and subject pool differences used in the present study could explain the absence of these genes from our profile. *CYP1B1 *is expressed to a greater degree in the females than in males and our data set is all male [64]. *SOD2 *gene expression declines with age [65]. The mean age of one of the studies reporting differential regulation of *SOD2 *was 27 years while the mean age of our study subjects is 46.5 years, which may explain why the *SOD2 *gene expression ratios between the groups in our study did not vary significantly.

A significant link has been established between smoking and the development of blood-borne cancers such as acute myelogenous leukemia (AML) and acute lymphocytic leukemia (ALL) [66,67]. Exposure to compounds derived from tobacco use is typically highest in the oral and nasal cavities, the laryngotracheobronchial tree, and the urinary system, putting these tissues at the greatest risk of developing tumors [68]. Nevertheless, given chronic exposure to carcinogens, blood tissues are likewise at an increased risk of carcinogenesis [69]. Sandler, *et al*., observed a clear dose response to smoking, with heavy smokers at the highest risk of developing leukemia [66]. The causative mechanism for this observed increase in leukemia among smokers is unknown. Our results identify highly relevant, differentially expressed genes that may serve as the basis for future experiments aimed at addressing the molecular etiology of AML and ALL in smokers. Moreover, these gene signals were detected in an easily obtainable sample of peripheral blood.

We found a correlation between tobacco use and increased expression of interferon-inducible genes in circulating leukocyte populations. Strong induction of interferon-responsive gene expression was seen in only a subset of tobacco-using subjects, arguing that interferon induction is not a direct effect of tobacco use. The mechanism of induction of these genes is not clear from our data. Previous studies have found a strong correlation between the parenchymal destruction associated with end-stage emphysema and the presence of interferon and interferon-inducible genes in the lung [60]. Intriguingly, 5 of the 6 subjects (83%) with a diagnosis of COPD in this study demonstrated the high-interferon response phenotype. Our observation of elevated peripheral interferon response gene expression may reflect a systemic manifestation of a destructive pulmonary inflammatory response. These observations may provide evidence of a systemic immune basis for smoking-related lung parenchymal destruction. Alternatively, the expression of interferon-responsive genes in the periphery may be secondary to the upper and lower respiratory tract infections to which smokers are prone.

Hyperclustering revealed 5 distinct, physiologically relevant gene groups in peripheral leukocytes affected by tobacco use *in vivo*. Furthermore, these gene groups belong to pathways and regulatory systems important to the etiology of smoking-related diseases. These novel results enhance our understanding of how tobacco use affects patterns of gene expression in leukocytes, and provide a starting point for elucidating the molecular mechanisms of tobacco-related neoplasia, atherosclerosis, and immune dysfunction. The hyperclustering visualization facilitated interpretation of microarray data by fusing the expression data with functional annotation derived through robust statistical methodology (GSA and GATHER) prior to cluster analysis. This technique is a visual representation that combines gene expression data and any form of additional annotation. Gene expression profiling of readily obtainable peripheral blood samples identified genes that regulate response to stress, macromolecular metabolism, transcription and signaling, interferon response, and cell death and resistance to apoptosis. This profile may identify some novel targets for therapeutic intervention for both smoking-related diseases and, potentially, for smoking cessation.

## Competing interests

The authors declare that they have no competing interests.

## Authors' contributions

PCC participated in design of the study, recruited subjects, processed samples, analyzed data, performed statistical analysis, and participated in manuscript preparation. BA recruited subjects, analyzed data, and participated in manuscript preparation. EGH processed samples, analyzed data, assisted in statistical analysis, and participated in manuscript preparation. JCS assisted in statistical analysis and manuscript preparation. REL participated in study design and coordination, and assisted in manuscript preparation. SM processed samples. JSP assisted in study design and data management. SSW participated in study coordination and recruited patients. AP assisted with manuscript preparation. GAS participated in study design and coordination, and data analysis. CP conceived of the study, participated in study design and coordination, performed data analysis, and participated in manuscript preparation.

## Pre-publication history

The pre-publication history for this paper can be accessed here:



## Supplementary Material

Additional file 1 Principle component analysis (PCA) of subject co-morbidities. PCA was performed using the combined significant gene list and visualized in the context of COPD, Diabetes, CAD class, and smoking status. As expected, the top component of variation is associated with smoking status. Additionally, it does not appear associated with the remaining variables. To formally test this hypothesis, the PC1 loadings were tested for association with each of the 4 clinical variables. Smoking status was found to be significantly associated with PC1 (p < 0.001). However, none of the remaining clinical variables were associated with the top component of variation (COPD p = 0.91; CAD p = 0.15; Diabetes p = 0.55) indicating that this gene list is not strongly associated with these disease states. Click here for file

Additional File 2 **Complete gene list of 109 genes identified by SAM and GSA**. Differentially expressed genes identified by SAM and GSA demonstrate the up-regulation of 34 genes and the down-regulation of 75 genes in subjects with high versus low plasma cotinine. The table includes the gene symbol, gene name, Genbank Accession ID, Agilent Probe ID and the mean fold change in gene expression in high *versus *low plasma cotinine subjects. Click here for file
