# Supplementary figures and images for: Tobacco use induces anti-apoptotic, proliferative patterns of gene expression in circulating leukocytes of Caucasian males
Source: BMC Med Genomics. 2008 Aug 18;1:38. doi: 10.1186/1755-8794-1-38 (PMC2531187; doi:10.1186/1755-8794-1-38)

**Cotinine Levels**

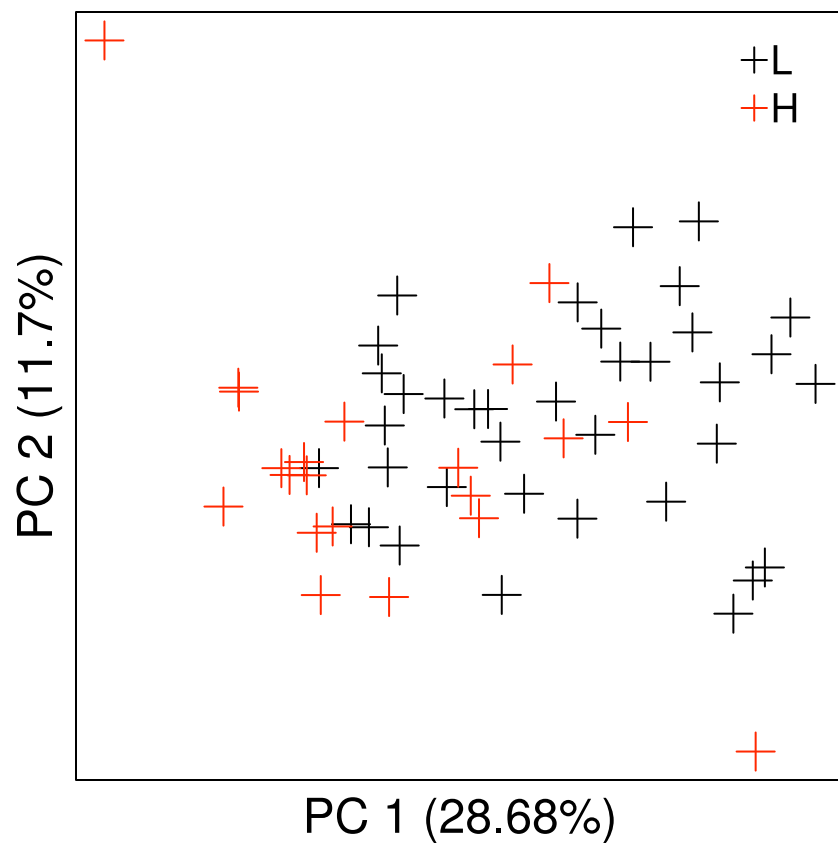

**COPD**

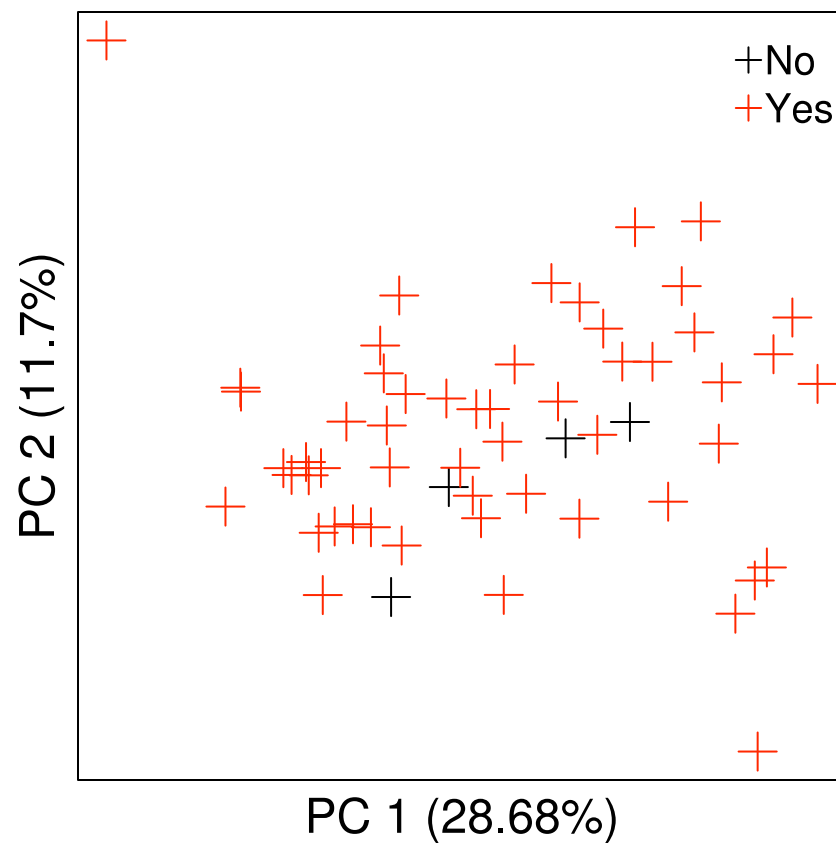

**CAD Classes**

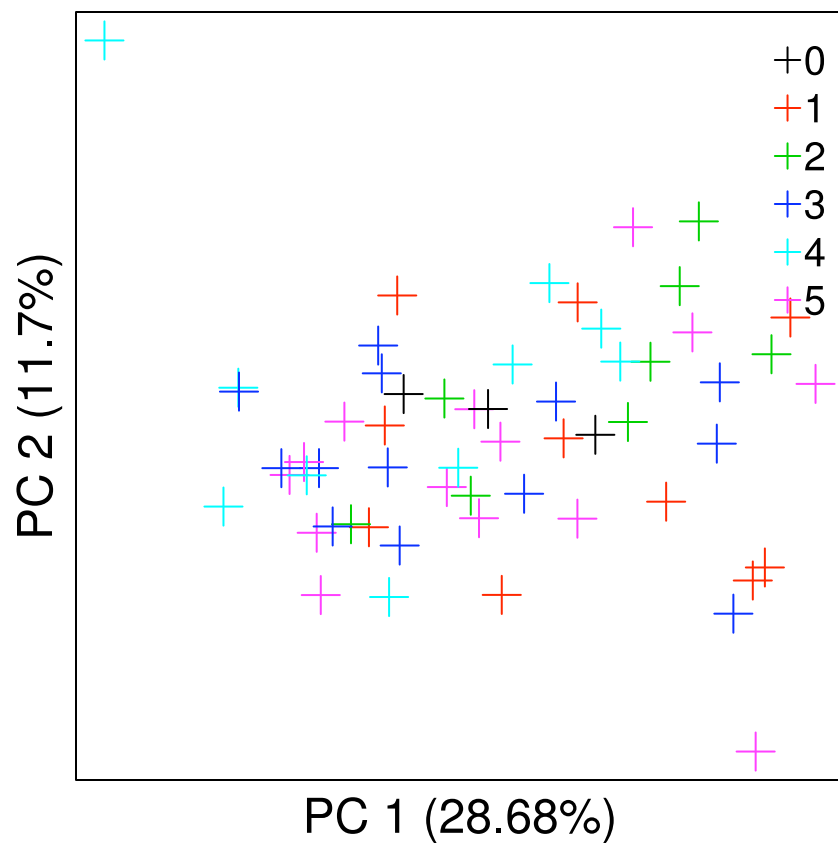

**Diabetes**

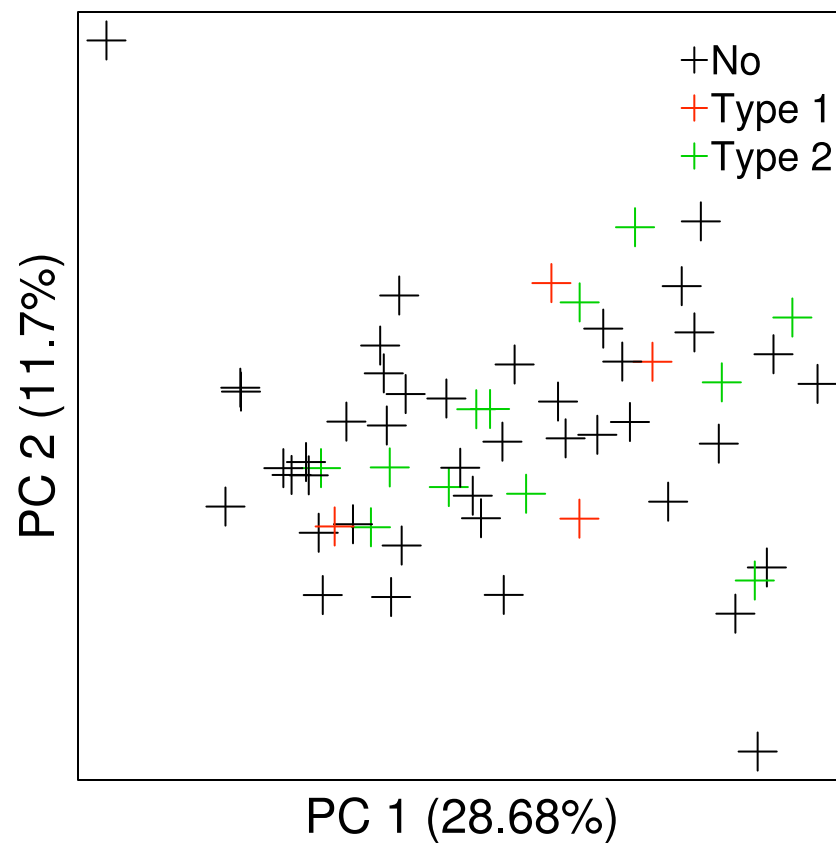

Supplement: Additional file 1 — Principle component analysis (PCA) of subject co-morbidities. PCA was performed using the combined significant gene list and visualized in the context of COPD, Diabetes, CAD class, and smoking status. As expected, the top component of variation is associated with smoking status. Additionally, it does not appear associated with the remaining variables. To formally test this hypothesis, the PC1 loadings were tested for association with each of the 4 clinical variables. Smoking status was found to be significantly associated with PC1 (p < 0.001). However, none of the remaining clinical variables were associated with the top component of variation (COPD p = 0.91; CAD p = 0.15; Diabetes p = 0.55) indicating that this gene list is not strongly associated with these disease states. [file 1755-8794-1-38-S1.pdf]
